# 

**Published:** 1848-03

**Authors:** 


*Pecuniary Present to Dr. Forbes.*—A circular has been sent to this country, inviting members of the profession to make subscriptions to make up the pecuniary losses which Dr. Forbes sustained in his "Commercial speculation" of carrying on the British and Foreign Medical Review. It strikes us that this appeal would with more propriety be made to the Homœopathists and Hydropathists, but, at all events, before sending our mite, we should be glad to be informed of the amount of Dr. F's income tax, and, also, whether in our own country there are any parallel instances of editorial sacrifices. If any of our readers are less scrupulous about the matter, they may remit their contributions to Dr. John C. Warren, of Boston, Mass.

---

*Rush Medical College.*—By an article in the Illinois and Indiana Medical and Surgical Journal, we learn that the Faculty of this flourishing Institution, at Chicago, Ill., have resolved to increase the number of profesorships to seven, by instituting a chair of Physiology and Pathology. This is done in accordance with the recommendation of the National Convention. The Faculty announce their readiness to extend the lecture term so soon as this measure is generally adopted by the medical schools of the West.

---

*Delegates to the American Medical Association.*—We perceive that delegates to the American Medical Association, which is to hold its annual meeting at Baltimore on the first Tuesday of May next, are being appointed in different States. We anticipate for the next meeting a much larger number of delegates than were convened last spring, and an occasion of still greater interest.

